# The Knowledge‐Practice Gap in Prescribing: A Study of Perceptual and Experiential Predictors at Asella Referral and Teaching Hospital, Ethiopia—A Cross‐Sectional Study

**DOI:** 10.1002/hsr2.73384

**Published:** 2026-10-14

**Authors:** Olana Daba, Tesfa Gebremeskel, Abdurrahman Kalu, Ayalneh Demissie

**Affiliations:** ^1^ Department of Pediatrics, College of Health Sciences Arsi University Asella Ethiopia; ^2^ Department of Public Health, College of Health Sciences Arsi University Asella Ethiopia

**Keywords:** brand drugs, ethiopia, generic drugs, knowledge‐practice gap, STROBE

## Abstract

**Background:**

Generic prescribing is a cornerstone of sustainable healthcare delivery, yet provider preference for brand‐name pharmaceuticals remains a widespread challenge in resource‐limited settings. This study evaluated brand‐name prescribing practices and identified independent predictor domains among healthcare prescribers at Asella Referral and Teaching Hospital (ARTH), Ethiopia.

**Methods:**

A facility‐based cross‐sectional study was conducted between August and October 2024, involving a census of 229 prescribers. Data were collected using structured, pretested questionnaires. Key domain scores were operationalized as continuous composite variables (1–5 Likert‐type scale) to maintain data integrity. A multivariable linear regression model was estimated to identify independent associates of brand‐prescribing practice following verification of classical regression assumptions.

**Results:**

Prescribers demonstrated high objective awareness of generic policies and drug efficacy (domain agreement of 76.0%); however, non‐adherence to generic‐favouring practices was reported by 54.2% of respondents, illustrating a marked knowledge‐practice gap. The overall linear regression model was statistically significant (*F* (6, 222) = 44.424, *p* < 0.001, Adjusted *R* ^2^ = 0.533). Positive Perception of Brand Drugs (standardized *β* = 0.416, *p* < 0.001) and Work Experience (standardized *β* = 0.398, *p* < 0.001) emerged as the only significant independent predictors. Policy knowledge, pharmaceutical promotion, and patient factors did not retain statistical significance.

**Conclusion:**

A clear divergence exists between regulatory policy knowledge and routine prescribing practices. Brand‐name prescribing demonstrated significant positive independent associations with internalized provider perceptions and clinical tenure. Hospital leadership and policymakers should complement policy dissemination with targeted interventions addressing provider perceptions and clinical habit formation.

**Abbreviations**
ARTH: Asella Referral and Teaching Hospital
EFDA: Ethiopian Food and Drug Authority
FDA: Food and Drug Administration (USA)
FMOH: Federal Ministry of Health (Ethiopia)
GPP: Good Prescribing Practices
IRB: Institutional Review Board
KAP: Knowledge, Attitude, and Practice
LMICs: Low‐ and Middle‐Income Countries
NHS: National Health Service (UK)
SDG: Sustainable Development Goals
SPSS: Statistical Package for the Social Sciences
UHC: Universal Health Coverage
WHO: World Health Organization

## 1 Introduction

### 1.1 Background/Rationale

The global escalation of pharmaceutical expenditures poses a significant threat to the sustainability of healthcare systems [1, 2]. To mitigate these costs, the WHO and regulatory bodies such as the FDA advocate for the use of generic medicines as bioequivalent, cost‐effective alternatives to innovator brands [3, 4]. In sub‐Saharan Africa, including Tanzania and Ethiopia, where out‐of‐pocket health expenditure remains disproportionately high, the economic benefit of transitioning to generic is critical for improving patient adherence and preventing catastrophic health spending [5, 6].

Despite the implementation of Good Prescribing Manuals, institutional formularies, and mandatory generic policies in Ethiopia [6, 7, 8], brand‐name drugs continue to dominate prescriptions. This phenomenon is often attributed to a persistent “perception gap” regarding the manufacturing quality and clinical effectiveness of generic drugs [9]. While high‐income health systems, such as the UK's National Health Service (NHS) have successfully integrated generic substitution through strong regulatory enforcement [10, 11, 12], many African health systems continue to struggle with irrational prescribing patterns [5, 8]. These ongoing struggles across sub‐Saharan African health systems are driven by systemic operational vulnerabilities. Chronic pharmaceutical supply chain volatility and frequent essential medicine stockouts force clinicians to default to whatever proprietary brands are available in local commercial pharmacies. Furthermore, regional regulatory bodies often lack the local bioequivalence testing infrastructure required to routine‐verify generic quality, fostering lingering skepticism among clinicians regarding bioequivalence and therapeutic equivalence. Concurrently, prescribers are subjected to intensive, targeted drug promotion by pharmaceutical representatives who exploit these doubts to build brand equity.

Effective prescribing requires strict adherence to rational principles [2, 9], yet vulnerable populations in low‐ and middle‐income countries remain underserved due to inflated drug costs driven by brand‐name reliance [11].

The WHO framework on essential medicines emphasizes that bioequivalence, reliable supply, and affordability are foundational pillars of Universal Health Coverage (UHC) [1, 13, 14, 15]. However, achieving equitable medicine access remains a persistent challenge across Eastern Ethiopia [14]. Emerging research indicates that prescribing is not merely a clinical transaction, but a complex behavioral outcome shaped by patient‐centered care dynamics [15] and heavily influenced by aggressive pharmaceutical marketing [16, 17]. In Ethiopia, promotional strategies deployed by pharmaceutical companies have been shown to directly shift physician prescribing patterns away from generics alternatives [16, 17]. This commercial influence often manifests as a systematic bias toward innovator brand‐name drugs, reinforced by direct manufacturer incentives and marketing mixes [17, 18, 19].

Furthermore, a pronounced “knowledge‐practice gap” presents a major hurdle to rational medicine use. While healthcare providers frequently demonstrate high theoretical knowledge regarding generic bioequivalence and essential drug policies [20, 21], their actual attitude and prescribing practices remain strongly biased toward brand‐name products [21, 22, 23]. This discrepancy is reinforced by entrenched perceptions that brand‐name medicines possess superior safety and efficacy profiles [24, 25]. Additionally, patient expectations and the interpersonal “discomfort” of declining patient requests for specific brands can lead clinicians to prescribe brand names against their better clinical judgment [26, 27]. For health sciences researchers, medical educators, and clinical trainees, understanding the structural and psychological drivers of this gap is vital for informing policy reform [28, 29]. Ultimately, reducing patient healthcare costs and improving health system efficiency depends on the successful regulation market entry and prescriber acceptance of generic medicines [30, 31, 32], supported by comprehensive public and clinician guidance [33].

While individual determinants of prescribing have been explored in isolation, no study in Ethiopia has simultaneously evaluated the independent contributions of brand perception, promotional intensity, prior clinical experience, professional knowledge, educational background, and patient factors within a multivariable framework. At Asella Referral and Teaching Hospital (ARTH), this knowledge‐practice gap and its independent predictors have not been quantitatively mapped.

### 1.2 Objectives

This study seeks to identify whether perceptual or experiential domains serve as the primary independent predictors of brand‐name drug preference among clinicians at ARTH.

## 2 Methods

### 2.1 Study Design

A facility‐based cross‐sectional study was conducted to assess the factors influencing brand‐name drug preferences among healthcare providers.

### 2.2 Setting

The study was conducted at **ARTH**, located in Asella town, 175 km southeast of Addis Ababa. Asella serves as the administrative center of the Arsi Zone, Oromia Region. This tertiary referral and teaching hospital serves a vast catchment area of approximately 3.5 million people and employs a total of 260 physicians. This academic environment is critical for assessing the “knowledge‐practice gap” among both seasoned clinicians and early‐career researchers [28, 29]. Data collection: August to October 31, 2024.

### 2.3 Participants

The Source population consists of the 260 physicians (including specialists, residents, and interns) and pharmacy professionals currently practicing at ARTH. From this source population, 231 prescribers were deemed eligible for the study after excluding non‐prescribing clinicians, staff on leave, and the principal investigator. The Study Population was therefore defined as all eligible prescribers actively engaged in medicine prescribing practice at ARTH, with 229 completing the survey. These participants were selected as the primary clinical decision‐makers who manage therapeutic choices under the influence of patient requests and pharmaceutical marketing [16, 26].

### 2.4 Variables

#### 2.4.1 Dependent Variable

The dependen**t** variable was preference for brand‐name drugs over generic alternatives [21].

### 2.5 Independent Variables

**Perceptual**—Beliefs on bioequivalence, perceived safety, and efficacy of generics [24, 25].

**Experiential**—Exposure to marketing mixes, company payments, and patient‐driven requests [17].

**Socio‐demographic**—Professional role, years of experience, and level of clinical training.

### 2.6 Data Sources/Measurement

Data were collected through a structured, pre‐tested, self‐administered questionnaire adapted from established WHO prescribing indicators [4] and existing literature on the Ethiopian “perception gap” [9, 22]. The tool was designed to measure whether the hospital's high level of theoretical knowledge [20, 21] translates into rational prescribing or is overridden by systematic bias toward brand‐name drugs [18, 19]. Two BSc‐level pharmacists were recruited as data collectors and received 1 day of intensive training on the study protocol and data collection ethics. To ensure data quality, the questionnaire was pre‐tested on 10% (*n *= 26) of the total sample size at the nearby Adama Hospital, which has a similar contextual background. Accordingly, the questioners were adjusted for consistency of the response and validity before implementation at ARTH. Before main data collection, a pre‐test demonstrated an overall pilot reliability of *α *= 0.834. In the full study sample (*N* = 229), the overall 21‐item measurement instrument exhibited high internal consistency (*α* = 0.872). Individual sub‐scale Cronbach's *α* values confirmed acceptable reliability across the main study domains: Prescribing Practice (*α* = 0.860), Brand Perception (*α* = 0.713), Knowledge (*α *= 0.678), Work Experience (*α* = 0.792), Promotional Activities (*α* = 0.656), and Patient Factors (*α* = 0.519). Data completeness and consistency were checked daily by the supervisor and principal investigators.

**Prescribing Practice:** The dependent variable was assessed using five Likert‐type‐scale questions regarding brand‐name drug use. To maintain data granularity and statistical power, a total composite score was calculated for each participant. Higher scores indicated a higher frequency of brand‐name prescribing.

**Knowledge and Perception:** *Knowledge* was assessed through three items focused on generic policy and therapeutic equivalence.

*Perception* was measured via five items regarding the effectiveness and safety of generic versus brand drugs. Both variables were maintained as continuous composite scores for the primary analysis.

### 2.7 Bias

To ensure the validity of findings:

**Anonymity:** Survey results are kept strictly confidential to mitigate social desirability bias regarding pharmaceutical industry interactions [17].

**Design:** The study focuses on the specific “perception gap” [9]; the study design isolates behavioral correlates of brand preference from clinical decision‐making factors.

**Study Size:** Census approach (*N* = 229) provided 53.3% explanatory power (*R* ^2^).

### 2.8 Statistical Analysis

Data were entered, validated, and cleaned using EpiData Software (Version 3.1) to ensure data integrity through double entry and automated validation checks. Cleaned datasets were exported to IBM SPSS Statistics for Windows, Version 27.0 (IBM Corp., Armonk, NY, USA) for statistical modelling and analysis.

Descriptive statistics were computed to summarize respondent sociodemographic characteristics and primary domain scores. Categorical variables were presented as frequencies and percentages, while composite Likert scale domain scores (ranging from 1 to 5) were operationalized and evaluated as continuous measures (mean ± standard deviation). To identify independent predictors of brand‐name prescribing practice, a multiple linear regression model was estimated. Multiple linear regressions were chosen because the primary dependent outcome—the prescribing practice composite score—is a continuous metric. All independent predictor domains (Perception of Brand Drugs, Work Experience, Pharmaceutical Promotion, Professional Knowledge, Educational Background, and Patient Factors) were included a priori based on established behavioral decision‐making frameworks. Before hypothesis testing, classical assumptions for multivariable linear regression were systematically evaluated:

1. Linearity: Evaluated and confirmed via bivariate scatter plots between individual continuous predictors and the dependent variable.
2. Independence of Residuals: Verified using the Durbin–Watson statistic, yielding a value of 2.179 (falling well within the acceptable non‐autocorrelation range of 1.5–2.5).
3. Normality of Residuals: Inspected visually via normal probability Q‐Q plots of standardized residuals, demonstrating an approximate normal distribution.
4. Homoscedasticity: Evaluated by plotting standardized residuals against standardized predicted values (ZRESID vs. ZPRED), confirming equal variance across fitted values.
5. Multicollinearity: Assessed using Variance Inflation Factors (VIF) and Tolerance metrics. All predictor VIF values ranged strictly between 1.049 and 2.026 (Tolerance > 0.490), well below the standard collinearity threshold of VIF > 5.0. 6. Influential Outliers: Screened using Cook's Distance (all values < 1.0) and leverage values to ensure no individual observation disproportionately influenced model parameters. For the multivariable linear regression model, both unstandardized coefficients (B with standard errors [SE]) and standardized beta weights (*β*) were calculated, alongside 95% confidence intervals (CI). Statistical significance was defined at a two‐tailed alpha level of *p* < 0.05.

## 3 Results

### 3.1 Response Rate and Sociodemographic Characteristics

A high response rate was achieved, with 229 prescribers completing the questionnaire out of the 231 source population.

Among 229 participants, 90.4% were male, and 80.8% had > 3 years of experience. Most were residents (55.0%), followed by specialists (26.2%), GPs (17.5%), and subspecialists (1.3%) see Figure 1 and Table 1.

**Figure 1:**
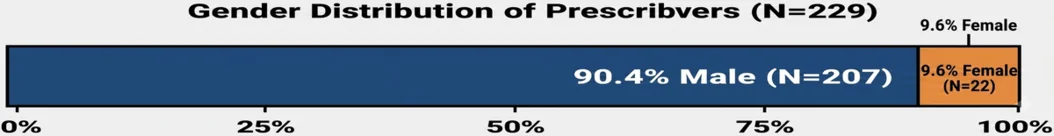
Gender distribution of prescribers (*N *= 229) at ARTH 2024.

**Table 1:** Sociodemographic profile of prescribers (N = 229) at ARTH 2024.

Variable	Category	*n*	%
Experience	< 1 year	8	3.5
	1–3 years	36	15.7
	> 3 years	185	80.8
Educational level	General practitioner	40	17.5
	Resident	126	55.0
	Specialist	60	26.2
	Subspecialist	3	1.3

### 3.2 Evidence of the Knowledge‐Practice Gap

Descriptive analysis of the primary domain continuous scores provided initial evidence of a substantial divergence between prescribers' knowledge of generic prescribing policies and their reported clinical practices (Table 2). The domain for Knowledge of Policy and Guideline Recommendations recorded the highest overall mean agreement, with an average of 76.0% of prescribers affirming their familiarity with generic policies and general generic drug efficacy. However, this high objective awareness did not translate into consistent reported practice. The domain for Prescribing Practice, measuring self‐reported adherence to generic‐favouring behaviors, demonstrated the highest average level of disagreement (54.2%). This contrast between policy knowledge and reported compliance illustrates the central knowledge‐practice gap in the study setting. Furthermore, provider perceptions regarding brand‐name drugs and external influencing factors presented as key continuous domains evaluated alongside policy adherence. Prescribers reported notable exposure to external factors—including Work Experience, Pharmaceutical Promotion, and Patient Pressure—with an average domain agreement of 54.7%. Perceptions toward Brand Drugs were mixed, showing a slight tendency toward disagreement (44.2% disagreement vs. 41.5% agreement). Table 2 summarizes the average agreement and disagreement percentages across all statements within each continuous domain.

**Table 2:** Domain analysis of the knowledge‐practice gap (*N* = 229) at ARTH 2024.

Domain	Avg. disagree (%)	Avg. agree (%)
Knowledge of policy & guideline recommendations	14.2	76.0
Influence of experience, promotion, & patient pressure	31.3	54.7
Perceptions toward brand drugs	44.2	41.5
Prescribing practice	54.2	36.1

### 3.3 Regression Diagnostics and Model Assumptions

Prior to hypothesis testing, classical assumptions for multiple linear regressions were formally evaluated. Independence of residuals was confirmed with a Durbin–Watson statistic of 2.179. Visual inspection of standardized residual scatter plots (ZRESID vs. ZPRED) and normal *Q–Q* plots confirmed homoscedasticity and approximate normality of error terms. Collinearity diagnostics demonstrated no problematic multicollinearity, with Variance Inflation Factors (VIF) ranging between 1.049 and 2.026 and all Tolerance values exceeding 0.490 across the six independent predictors.

### 3.4 Multiple Linear Regression Analysis

The overall multiple linear regression model was statistically significant (*F* (6, 222) = 44.424, *p* < 0.001), demonstrating a strong linear relationship (*R* = 0.739) and explaining 53.3% of the variance in prescribing practice (adjusted *R* ^2^ = 0.533, *R* ^2^ = 0.546, standard error of the estimate = 0.734).

The multiple linear regression model was structured as follows: *Y*_Brand Prescribing_ = B_0_ + B_1_(Perception) + B_2_(Brand Experience) + B_3_(Promotion) + B_4_(Knowledge) + B_5_(Education) + B_6_(Patient Factors) +ℇ, where ϵ‐represents the unobserved residual error term. Table 3 summarizes the multiple linear regression model evaluating predictors of brand prescribing practices among prescribers at ARTH during August–October 2024. Although multiple domains demonstrated high descriptive prevalence, only two retained statistically significant independent associations with brand‐name prescribing practice: Positive Perception of Brand Drugs and Work Experience. Positive Perception of Brand Drugs emerged as the strongest independent predictor, exhibiting a highly significant positive association with brand‐name prescribing (β = 0.416, *p* < 0.001). This indicates that subjective provider beliefs favouring brand‐name products serve as a primary independent associate of non‐generic prescribing behavior. The second strongest independent predictor was Work Experience, which also demonstrated a significant positive association with brand‐name selection (*β* = 0.398, *p* < 0.001), indicating that longer professional tenure correlates with a solidifying of brand‐prescribing habits. Conversely, while Knowledge of Policy and Guideline Recommendations was descriptively high (76.0%), it was not a statistically significant independent predictor in the multivariable model (*p* > 0.05). Similarly, continuous domain measures for Pharmaceutical Promotion and Patient Factors did not achieve statistical significance as independent predictors when controlling for other model variables, suggesting an indirect pathway where external influences shape internalized provider perceptions over time.

**Table 3:** Multiple linear regression model predicting prescribing practice score (*N* = 229).

Predictor	*B*	(SE)	95% CI	*β*	t‐statistic	*p*‐value	Tolerance	VIF
(Constant)	−0.319	0.357	(−1.023, 0.386)	—	−0.891	.374	—	—
Positive perception (Per)	**0.558**	**0.081**	**(0.398, 0.717)**	**0.416**	**6.891**	**< 0.001**	0.562	1.780
Work experience (Expre)	**0.359**	**0.058**	**(0.245, 0.474)**	**0.398**	**6.179**	**< 0.001**	0.494	2.026
Pharmaceutical promotion (Promo)	0.071	0.062	(−0.050, 0.193)	0.060	1.158	.248	0.762	1.312
Knowledge level (Knowledge)	0.048	0.058	(−0.067, 0.163)	0.038	0.817	.415	0.953	1.049
Education level (Edu)	0.052	0.073	(−0.092, 0.195)	0.033	0.712	.477	0.930	1.075
Patient factors (PatientF)	−0.055	0.079	(−0.211, 0.101)	−0.041	−0.693	.489	0.585	1.710

*Note:* Bold values denote statistically significant independent predictors (*p* < .001).

*Model Summary*: Dependent variable = Prescribing practice (Practice). *R* = 0.739, R^2 = 0.546, Adjusted R^2 = 0.533, Std. error of estimate = 0.734. Model ANOVA: F (6, 222) = 44.424, *p* < 0.001. Residual independence: Durbin–Watson = 2.179.

Table 3 summarizes the multiple linear regression model evaluating predictors of brand prescribing practices among prescribers at ARTH during August–October 2024.

## 4 Discussion

An examination of prescribing behavior at ARTH during August–October 2024 reveals a complex challenge: a substantial knowledge‐practice gap regarding generic drug prescribing. This phenomenon is supported by the descriptive findings (Table 2), which demonstrate a clear divergence between prescribers' policy awareness and their routine clinical habits. Specifically, while prescribers exhibited a high level of familiarity with generic policies and regulatory recommendations—with the Knowledge of Policy and Guideline Recommendations domain achieving the highest mean agreement (76.0%)—their actual prescribing practices did not consistently reflect this knowledge, as evidenced by the high average disagreement (54.2%) observed in the Prescribing Practice domain. This finding aligns with earlier reports from diverse health system settings [26, 27, 28, 29] and confirms that high policy awareness alone does not guarantee rational generic prescribing.

The primary objective of this discussion is to move beyond this descriptive divergence and interpret the multivariable linear regression findings, which identify Positive Perception of Brand Drugs and Work Experience as the primary independent factors associated with this knowledge‐practice gap. In the multivariable linear regression analysis, Positive Perception of Brand Drugs emerged as the strongest independent predictor of brand‐name selection, supporting earlier literature [13, 14, 15] that links reliance on brand‐name products to underlying perceptions regarding generic quality and bioequivalence. Because theoretical knowledge was not a primary driver in the multivariable model, the data suggest that clinical decision‐making in this context is closely tied to subjective provider perceptions that may override formal policy awareness.

The second strongest predictor, Work Experience, indicates that brand‐prescribing patterns are strongly associated with accumulated years in clinical practice. This observation aligns with global literature on prescribing routines and the cumulative effect of long‐term exposure to clinical environments [19, 20, 21, 22, 23], where prescribing habits become firmly established over time.

A key nuance in this study is that both Pharmaceutical Promotion and Patient Factors did not retain statistical significance in the final multivariable model, despite their high descriptive prevalence (54.7% agreement). This suggests that external promotional influences and patient requests may operate indirectly by shaping a prescriber's internalized brand perception over the course of their career. Consequently, institutional and regulatory interventions should focus on addressing internal belief systems and clinical attitudes rather than relying solely on passive policy distribution or targeting external promotional factors in isolation.

Our findings provide novel insights into the specific operational realities that perpetuate the knowledge‐practice gap in Ethiopian tertiary healthcare. Prior studies in Sub‐Saharan Africa have often attributed lower generic adoption primarily to a deficit in provider knowledge. However, by simultaneously modelling multiple perceptual, behavioral, and organizational factors, our results demonstrate that brand perception and accumulated clinical experience exert a substantially stronger independent influence on prescribing decisions than theoretical knowledge alone. This suggests that clinician behavior at ARTH is closely linked to empirical brand trust and clinical familiarity—likely developed as an adaptive heuristic against regional supply chain volatility and historical concerns over generic bioequivalence. Addressing these prescribing patterns will therefore benefit from structural health‐system interventions, such as strengthening local regulatory quality assurance, improving bioequivalence transparency, and implementing institutional counter‐detailing, rather than relying exclusively on passive educational campaigns.

### 4.1 Strengths and Limitations

Strengths: The primary strength of this study lies in its robust explanatory power; the regression model achieved an Adjusted R2 of 0.533, lending high credibility to the identified predictive factors. The study successfully moved beyond descriptive data to distinguish between factors that are merely prevalent and those that are statistically predictive, offering highly actionable insights for policy formulation. Furthermore, the comprehensive data collection across knowledge, perception, and influence allowed for a direct, quantitative assessment of the knowledge‐practice gap.

### 4.2 Limitations

The study's principal limitation is its cross‐sectional design, which captures associations at a single point in time and therefore cannot establish temporal sequencing or causality. Consequently, reverse causality cannot be ruled out; it is plausible that prescribers who routinely select brand‐name drugs construct favorable post‐hoc attitudes toward them to rationalize their established clinical habits (justification bias), rather than brand perceptions acting strictly as an antecedent determinant of practice.

Additionally, findings are subject to self‐report bias (such as social desirability), meaning prescribers may have underreported influences that are professionally undesirable (e.g., promotional trust) or overestimated policy knowledge. Finally, the study is restricted to a single tertiary institutional setting (ARTH during August–October 2024), which limits the generalizability of the findings to diverse primary care or private sector practices.

**Interpretation:** Brand‐prescribing habits solidify with professional tenure (inertia). Regulatory efforts must focus on internal belief systems (Academic Detailing) rather than just external mandates.

**Generalizability:** Findings are applicable to similar tertiary teaching hospitals in low‐income settings.

## 5 Conclusion and Recommendations

## 6 Conclusions

This study demonstrates that internalized brand perception and clinical tenure are the primary independent predictors of brand‐name prescribing behavior among clinicians at ARTH, exhibiting a stronger relative statistical association than formal policy knowledge. In the multivariable linear regression model, reliance on brand‐name drugs was most strongly associated with prescribers' positive perception of brand‐name pharmaceuticals (*β *= 0.416, *p* < 0.001) and accumulated work experience (*β* = 0.398, *p* < 0.001). Despite high objective awareness of generic prescribing policies (76.0%), clinical practice remains inconsistent with national and international guidelines, suggesting that experiential habits and subjective beliefs may temper the practical application of policy knowledge. Ultimately, these findings highlight that narrowing the hospital knowledge‐practice gap requires strategies beyond administrative mandates alone. Strengthening rational prescribing in teaching hospital settings necessitates a shift toward addressing the specific perceptual and experiential factors that reinforce brand‐name preference over time.

## 7 Recommendations

To address the identified knowledge‐practice gap, institutional interventions should transition from passive information dissemination to targeted behavioral and structural strategies:

1. Addressing perceptual bias (*β* = 0.416): Hospital leadership should implement peer‐led Academic Detailing programs. Rather than relying solely on policy memos, trained clinical pharmacists or senior colleagues should conduct structured, one‐on‐one outreach to present empirical bioequivalence and quality‐assurance data, directly addressing subjective perceptions regarding brand‐name superiority.
2. Disrupting experiential inertia (*β* = 0.398): Given that brand‐prescribing tendencies correlate with clinical tenure, the hospital should incorporate “Generic‐by‐Default” mechanisms within paper‐based order sets and emerging Electronic Health Record (EHR) modules. Requiring explicit justification to opt out of generic formulation prompts a conscious decision, disrupting habituated prescribing routines.
3. Institutional Audit and Feedback: A structured Clinical Audit and Feedback mechanism should be established. Providing prescribers with regular, peer‐benchmarked feedback on generic prescribing rates leverages social and professional norms to encourage alignment with institutional guidelines.
4. Managing Commercial Influence: Access of pharmaceutical sales representatives to clinical personnel—particularly medical residents and junior prescribers—should be systematically regulated to minimize early exposure to promotional messaging that may shape brand‐positive perceptions over a clinician's career.

## Author Contributions

**Olana Daba:** conceptualization, investigation, writing – original draft, methodology, software, formal analysis, project administration, data curation, resources. **Tesfa Gebremeskel:** methodology, validation, writing – review and editing, supervision. **Abdurrahman Kalu:** supervision, validation, methodology, writing – review and editing. **Ayalneh Demissie:** methodology, validation, supervision, writing – review and editing.

## Funding

The authors have nothing to report.

## Ethical Statement

The study was conducted in accordance with the Declaration of Helsinki, and approved by the Institutional Review Board of Arsi University (protocol code A/CHS/RC/103/2024 and August 2024).

## Consent

Informed consent was obtained from all subjects involved in the study.

## Conflicts of Interest

The authors declare no conflicts of interest.

## Transparency Statement

The lead/corresponding author, **Dr. Ayalneh Demissie**, affirms that this manuscript is an honest, accurate, and transparent account of the study being reported; that no important aspects of the study have been omitted; and that any discrepancies from the study as planned (and, if relevant, registered) have been explained.

## Data Availability

All data generated or analyzed during this study are included in this published article and its supplementary information files.
